# FlexiChip package: an universal microarray with a dedicated analysis software for high-thoughput SNPs detection linked to anti-malarial drug resistance

**DOI:** 10.1186/1475-2875-8-229

**Published:** 2009-10-15

**Authors:** Nicolas Steenkeste, Marie-Agnès Dillies, Nimol Khim, Odile Sismeiro, Sophy Chy, Pharath Lim, Andreas Crameri, Christiane Bouchier, Odile Mercereau-Puijalon, Hans-Peter Beck, Mallika Imwong, Arjen M Dondorp, Duong Socheat, Christophe Rogier, Jean-Yves Coppée, Frédéric Ariey

**Affiliations:** 1 Laboratoire d'épidémiologie moléculaire, Institut Pasteur du Cambodge, 5 bd Monivong, BP 983, Phnom Penh, Cambodia; 2 Institut Pasteur, Génopole, Plate-forme Puces à ADN, F-75015 Paris, France; 3 Amunix, 500 Ellis Street, Mountain View, CA 94043, USA; 4 Institut Pasteur, Génopole, Plate-forme Génomique, F-75015 Paris, France; 5 Immunologie Moléculaire des Parasites, Institut Pasteur de Paris, Paris, France; 6 Swiss Tropical Institute, Socinstrasse 57, CH 4002 Basel, Switzerland; 7 Faculty of Tropical Medicine, Mahidol University, Bangkok, Thailand; 8 Center for Clinical Vaccinology and Tropical Medicine, Oxford, UK; 9 National Center for Parasitology, Entomology and Malaria Control, Phnom Penh, Cambodia; 10 IMTSSA, Unité de Recherche en Biologie et Epidémiologie Parasitaires (URBEP), Marseille, France

## Abstract

**Background:**

A number of molecular tools have been developed to monitor the emergence and spread of anti-malarial drug resistance to *Plasmodium falciparum*. One of the major obstacles to the wider implementation of these tools is the absence of practical methods enabling high throughput analysis. Here a new Zip-code array is described, called FlexiChip, linked to a dedicated software program, which largely overcomes this problem.

**Methods:**

Previously published microarray probes detecting single-nucleotide polymorphisms (SNP) associated with parasite resistance to anti-malarial drugs (ResMalChip) were adapted for a universal microarray FlexiChip format. To evaluate the overall sensitivity of the FlexiChip package (microarray + software), the results of FlexiChip were compared to ResMalChip microarray, using the same extension probes and with the same PCR products. In both cases, sequence results were used as gold standard to calculate sensitivity and specificity. FlexiChip results obtained with a set of field isolates were then compared to those assessed in an independent reference laboratory.

**Results:**

The FlexiChip package gave results identical to the ResMalChip results in 92.7% of samples (kappa coefficient 0.8491, with a standard error 0.021) and had a sensitivity of 95.88% and a specificity of 97.68% compared to the sequencing as the reference method. Moreover the method performed well compared to the results obtained in the reference laboratories, with 99.7% of identical results (kappa coefficient 0.9923, S.E. 0.0523).

**Conclusion:**

Microarrays could be employed to monitor *P. falciparum *drug resistance markers with greater cost effectiveness and the possibility for high throughput analysis. The FlexiChip package is a promising tool for use in poor resource settings of malaria endemic countries.

## Background

Anti-malarial drugs play a pivotal role in malaria control, but a limited number of new drugs are under development. Resistance of malaria parasites to commonly used anti-malarial drugs is also a global challenge. Thus, there is a need to optimize the use of existing treatments and to monitor the emergence and the spread of drug resistant malaria parasites, in particular *Plasmodium falciparum*, which is responsible for the vast majority of malaria deaths [1-4]. Typing the known genetic drug resistance markers is among the strategies currently used for monitoring the resistance of *P. falciparum*. Single nucleotide polymorphisms (SNP) related to anti-malarial drug resistance include five major genes: *Pfdhfr *and *Pfdhps *for pyrimethamine and sulphadoxine resistance, *Pfcrt *and *Pfmdr1 *for chloroquine resistance and recently, but not yet confirmed by field studies, *serca/atpase6 *for artemisinin resistance. Different molecular tools have been developed, including the PCR-RFLP method [5-8], real-time PCR for assessing gene copy number [9], sequence analysis [10], the heteroduplex tracking assay [11], and PCR-amplification of the SNP containing fragments followed by single base extension (SBE) of an elongation primer with fluorescent ddNTP's [12].

**Figure 1:**
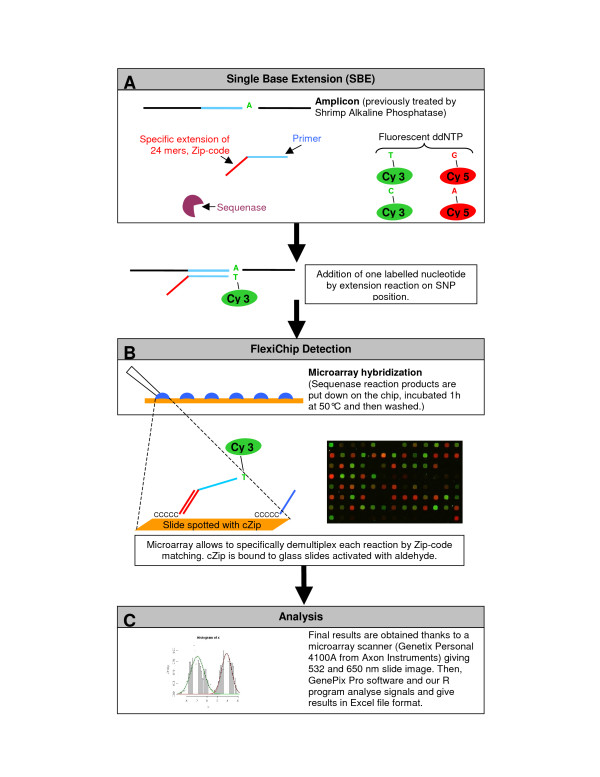
**Schematic representation of the FlexiChip analysis method**. A) SNPs are detected by Single Base Extension (SBE) using Sequenase and ddNTP labelled with Cyanine 3 or 5 using a specific probe that hybridizes one nucleotide upstream of the SNP site. B) Products of the SBE reaction are hybridized on FlexiChip by their Zip-code oligonucleotides. After washing and drying, the slides are scanned at two wave lengths. C) Analysis algorithm is based on a mixture model and allows accurate SNP identification. The results are stored in Excel file.

DNA microarray-based SNP genotyping has been importantly developed over the past recent years. Surveying SNPs is an important tool in epidemiological studies on parasite resistance, but the currently available methods to identify resistance all have important drawbacks, including a limited focus on only the five mentioned genes and absence of a high throughput format. Several systems have been proposed [13,14], mainly based on PCR-amplification of the SNP containing fragments followed by SBE of an elongation primer with fluorescent ddNTP's [15]. Recently, a genotyping array called ResMalChip has been developed to monitor 34 SNPs in five genes of *P. falciparum *that either confer or increase resistance to anti-malarial drugs [16]. The ResMalChip method also has two major drawbacks. First, the content of the microarray is designed only for a specific objective (typing SNPs related to resistance) in a specific organism. Therefore, the use of this microarray for surveying other SNPs in any other gene or organism requires a new array design and production. This implies a large number of tests to adapt the system to new markers. The design of the capture oligonucleotides must be specific of the elongation primers and the experimental conditions of hybridization must be compatible with all the couples of capture oligo-elongation primers. Moreover, no standard software has been developed for data analysis. A new Zip-code array, called FlexiChip, associated with a dedicated software has been designed to address these problems (see Figure 1) [17]. This array contains oligonucleotides (Zip-code) that are not complementary to any sequence in any known organism and have been designed to have the same thermodynamic properties. Therefore, various assays can be performed using a single protocol from SNP discovery to hybridization. The target probes contain the Zip-codes complementary sequences linked to the elongation primers. FlexiChip can in principle be used to test any SNP. Furthermore, an analysis algorithm based on a mixture model and allowing accurate SNP identification has been developed. This algorithm does not require any prior threshold determination and provides results in a simple Excel file format. To evaluate the overall sensitivity of the FlexiChip package (microarray + software), ResMalChip and FlexiChip data sets were analysed with this software and tested against sequence analysis. FlexiChip results were then compared to results obtained with ResMalChip using the same PCR products. For a set of 50 field isolates, FlexiChip results were also compared to those obtained in another molecular laboratory (MORU) acting as external quality control.

## Methods

### Clinical *P. falciparum *samples and DNA extraction

As part of the activities related to the assessment of drug efficacy in uncomplicated malaria in Cambodia, 263 *P. falciparum *isolates were collected from consenting patients from 2001 to 2004. Blood samples were kept at -20°C at the Institut Pasteur du Cambodge until use. DNA was extracted from blood samples using QIAmp^® ^DNA Mini Kit (Cat. No. 51306, QIAgen^®^, Germany), according to the manufacturer's procedure. All studies were conducted following good clinical practice, and ethical clearance was obtained from the National Ethical Comity of Cambodia.

### Microarray design

#### Zip code design and microarray production

A Zip-code as used in the present study is defined as an artificial sequence composed of 24 bases. All the Zip-codes have similar melting temperature (Tm) values. A set of 96 Zip-code of 24 mer oligonucleotides has been designed using a dedicated algorithm developed in Visual Basic (Microsoft^®^) (see Table S1 in Additional file 1). They were tested to avoid self-pairing and hairpin formation (FastPCR, Institute of Biotechnology, University of Helsinki [18]). Reverse complement oligonucleotides (cZip) were synthesized with an amino C-7 linker at the 3' end used for its attachment to the slide. Then cZip were spotted onto aldehydesilane coated slides with a 12-well format (AL MPX slides, Schott) using a VersArray ChipWritterPro system (Bio-Rad Laboratories, Hercules, CA). For spotting the cZip were resuspended at 50 micromolar in Phosphate buffer. FlexiChip spotting pattern of the 96 cZip with Cy3, Cy5 anchor prelabeled oligonucleotide and six negative controls is presented in additional figure 2A. Each oligonucleotide was spotted in triplicate. A total of 12 independent hybridizations can be performed in parallel on a single slide. ResMalChip arrays were produced as described in [16].

**Figure 2:**
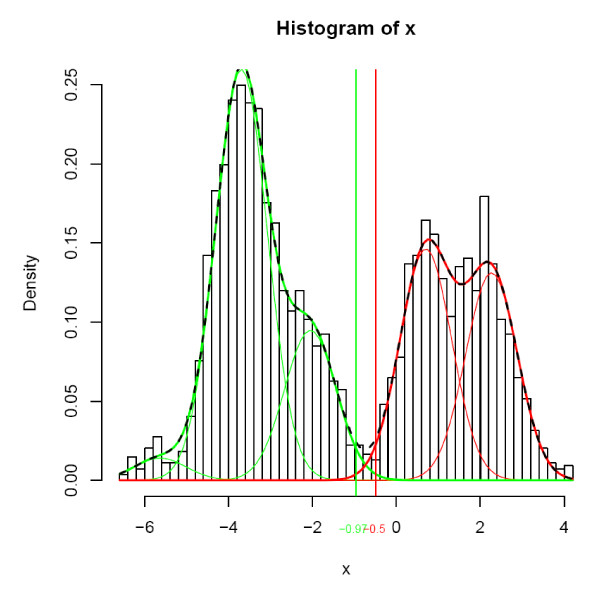
**The mixture model (FlexiChip)**. "Green": Gaussian components of the "green" class, "red": Gaussian components of the "red class", thick "green": prior conditional probability density function f(x/ω_1_), thick "red": prior conditional probability density function f(x/ω_2_), dashed black: mixture density function f(x) = f(x/ω_1_)P(ω_1_) + f(x/ω_2_)P(ω_2_), "green" vertical line: lower limit of ω_0_, "red" vertical line: upper limit of ω_0_

#### Microarray validation

To evaluate possible cross-hybridization between Zip-codes, each Zip-code associated with its primer was labelled using the SBE protocol (see below) and hybridized one by one on the microarray. Cross-hybridization was considered as significant when fluorescent average signal intensity of non tested Zip-code spots was above 10% of the average positive signal of the tested Zip-code spot. This was observed for only two spots out of 96 and the corresponding two Zip-codes were discarded from the analysis (see Table S1 in Additional file 1).

### Microarray protocol (Figure 1)

#### DNA amplification to genotype SNPs associated with anti-malarial drug resistance

Ten nested PCR were used to amplify DNA sequences of genes including SNPs associated with drug resistance as reported previously[16]: *pfmdr1 *N86Y, Y184F, S1034C, N1042D and D1246Y (two nested PCR); *pfcrt *C72S, K76T, H97Q, T152A, S163R, A220S, Q271E, N326D/S, I356L/T and R371I (five nested PCR); *pfdhfr *A16V, N51I, C59R, S108N/T and I164L (one nested PCR); *pfdhps *S436A, A437G, K540E, A581G, A613T/S, I640F, and H645P (one nested PCR); and *pfATPase6 *S538R, Q574P, A623E, N683K, and S769N (one nested PCR).

#### Single base extension, SBE

Remaining free dNTP's were removed using a shrimp alkaline phosphatase (SAP). Briefly, 5 μl of those ten nested PCR products were mixed to 2 U of SAP (Amersham Biosciences, Freiburg, Germany) and incubated for 1 h at 37°C. From each sample, two reactions were performed using two combinations of Cy3 and Cy5 labelled ddNTP's (Perkin Elmer, Schwerzenbach, Switzerland). Sequenase (Termipol^®^, Solis, Tartu, Estonia) extension reaction, reaction mixture and final denaturation were done for ResMalChip and FlexiChip as described by Crameri *et al *[16]. Extended primers with cyanine labelling were hybridized onto the microarray. With this experimental design on FlexiChip, two samples can be processed per spotting area. As 40 positions are needed per sample, one set of extension primers can be associated with Zip-codes 1 to 40 while the second set can be associated with Zip-codes 49 to 88 (remaining positions 41 to 48 and 89 to 96 were not used).

#### Chip hybridization

Briefly, extended primers associated with a Zip-code were resuspended in 6 μl of 20 × SSC (1× SSC = 0.15 M NaCl, 0.015 M sodium citrate, pH 7.2) and hybridized on the array. Microarrays were then incubated during 60 min at 50°C, in a humid chamber and subsequently washed in 2 × SSC and 0,2% SDS for 20 min and in 2 × SSC for 20 min. Microarrays were spun 5 min at 3000 g to dry. During hybridization, extended primers linked with their specific Zip-code were hybridized on the FlexiChip cZip pattern (see Table S2B in Additional file 2).

#### Data acquisition

Hybridized microarrays were scanned at 635 nm and 532 nm using an Axon 4100A fluorescence scanner (Axon, Bucher Biotec AG, Basel, Switzerland) and Axon GenePix^® ^Pro (version 6.0) software. The PMT (photomultiplier tube) was 550 at 532 nm and 500 at 635 nm.

### Data analysis and allele identification

All the data analyses were performed using the R software [19] and packages. The allele identification algorithm was written in R. It was applied independently on each array. The aim of this algorithm is to classify each spot of the array in either one of the "green", "red", or "indeterminate" classes, and then convert the spot colour into the corresponding SNP sequence. ResMalChip and FlexiChip raw data were first corrected for background using the limma package [20] (version 2.12.0) according to a two-step procedure. A modified version of the "movingmin" option of the background correction function ("called "bgCorrect") was first applied to the data. This option smoothes the background on the basis of a 3 × 3 moving window. But unlike the original version, the modified version does not substract the smoothed background. Then the normexp procedure was applied. According to this procedure, the observed signal is modeled as the convolution of a true signal and a background one, where the true signal follows an exponential distribution and the background follows a Gaussian distribution.

This two-step process was derived because of a high background level observed with respect to the signal, especially on ResMalChip data. Spots that still had a signal to noise ratio lower than one after background correction were flagged "bg" (where "bg" stands for "background").

Data from negative and positive control spots were then excluded from the data set. An intensity threshold I_T _was computed on the remaining spots for each slide as the median of pooled "red" and "green" intensities. A log2 ratio of the "red" intensity over the "green" intensity was computed for each of the 1440 (three replicates of 40 SNPs spots for 12 samples) remaining spots.

A two-component Gaussian mixture model was fitted differently to the ResMalChip and FlexiChip datasets. For the ResMalChip dataset, a two-component Gaussian mixture model was computed using the Mclust function from the mclust package [21] with the *modelNames *parameter set to "E" (Gaussian functions with same variance). These two estimated Gaussian functions are estimates of the conditional prior probability functions f(x/ω_1_) and f(x/ω_2_) that describe the distribution of log ratios within the classes ω_1 _and ω_2 _(these two classes are respectively associated with "green" and "red" spots). For the FlexiChip dataset, the model was built in two steps. A first optimal mixture model was computed using the Mclust function with default parameters (modelNames = c("E","V")). In most cases a three or more components mixture model was obtained. To get a two-component mixture model these components were grouped according to the sign (positive or negative) of their mean and a mixture model was derived from each of these two groups. These two "sub"-models were then used as estimates of the conditional prior probability functions (see Figure 2).

The remaining of the base-calling algorithm was then identical for the datasets of both chips. Conditional posterior probabilities P(ω1/x) and P(ω2/x) were computed according to the Bayes theorem: P(ω_i_/x) = f(x/ω_i_). P(ω_i_)/[f(x/ω_1_). P(ω_1_) + f(x/ω_2_). P(ω_2_)], i = 1,2

A third class called ω_0 _was created between ω_1 _and ω_2_. Its boundaries were defined using a tunable parameter called ambiguity rejection threshold and denoted C_r_. This class contained data from spots that had a probability lower than C_r _of belonging to one of the "red" and "green" classes and was used to exclude data having a low probability of good classification, *i.e*. lower than C_r_.

Each spot on the array was first classified within one of the "green" (ω_1_)/"red" (ω_2_)/rejection (ω_0_)/weak signal/background (bg) classes according to the following decision rules:

• P(ω_1_/*x*) > P(ω_2_/*x*) and P(ω_1_/*x*) > C_r _and I_S _> I_T _and bg = FALSE → d(*x*) = ω_1_

• P(ω_2_/*x*) > P(ω_1_/*x*) and P(ω_2_/*x*) > C_r _and I_S _> I_T _and bg = FALSE → d(*x*) = ω_2_

• max(P(ω_i_/*x*)) ≤ C_r_, i = 1,2 and I_S _> I_T _and bg = FALSE → d(*x*) = ω_0_

• I_S _> I_T _and background = TRUE → d(*x*) = bg

• I_S _≤ I_T _→ d(*x*) = weak signal

where *x *is the log ratio associated with the spot, d(*x*) is the decision associated with *x*, I_SR _and I_SG _are respectively the "red" and "green" intensities measured on the spot, and I_S _= max(I_SR_, I_SG_) is the maximum of both intensities for this spot.

A final decision was taken for each SNP on the basis of its three replicate spots as follows: if at least two of the three replicates were belonging to the same class the SNP was associated with this class, otherwise it was declared "indeterminate" and no further interpretation was performed.

Allele identification was done using a pre-defined table that describes the expected signal for each allele of each SNP (see Table 1). This table was fully derived from the design of the experiment. As an example, according to this table a "red" signal (Cy5) is expected for spots associated with the RES16 SNP if the allele in the studied sample is a mutant, and a "green" signal (Cy3) otherwise. Three possible scenarios are encountered depending on the number of different probes that were associated with the SNP. In the first case, SNPs were represented by only one probe, meaning that only two different alleles were known for them. This was the most general case. Then, for SNPs that had been classified in ω_1 _or ω_2_, allele identification came straight from table 2. If a field sample was studied using FlexiChip or ResMalChip and the hybridization signal for the RES16 SNP was found to belong to the "red" class, the *Pfdhfr *gene from this sample was identified as mutant at position 16. The second scenario refers to SNPs that had only two known alleles but were represented by two different probes on the slide, in order to strengthen the identification process. Then, if one of the probes was classified as "weak signal" or "bg", the other probe result was taken into account. If both probe signals were valid (d(*x*) = ω_1 _or d(*x*) = ω_2_), the coherence between the probes was checked and in case of conflicting results the SNP was declared "indeterminate". The last scenario refers to the situation where more than two different alleles for a given SNP exist. Thus, three or four different alleles must be discriminated with two colours only. For these particular SNPs, two different probes were designed and the corresponding targets were labelled with two different combinations of Cy3 and Cy5 labelled ddNTP's, as explained in the experimental protocol section. For example, the position 108 on gene *Pfdhfr *is represented by two probes on the array, RES108 and RES108B. The first probe allows to distinguish between the wild type allele and either mutantA or mutantB. The second probe makes the difference between the mutantA and either wild type or mutantB. In such a case, allele identification was resolved according to the combination of both probe results. If one or both probe signals were classified "weak signal", "bg" or "indeterminate", the SNP was declared "indeterminate", otherwise it was determined according to Table 1. Mutually exclusive results for such two complementary probes led the associated SNP to be declared "indeterminate". As an example, this would be the case for the SNP RES108, if both probes gave "red" signals.

**Table 1:** Expected spot signals for SNP positions processed by the algorithm (Results are given as a comparison with 3D7 genotype; WT = Wild type, MUT = Mutation)

**Gene Name**	**SNP code**	**SNP name**	**Cy3 "green" signal**	**Cy5 "red" signal**	**Sequence**
*Pfdhfr*	RES16	16	WT	MUT	*
*Pfdhfr*	RES51	51	WT	MUT	*
*Pfdhfr*	RES59	59	WT	MUT	*
*Pfdhfr*	RES108	108	MutA or MutB	WT	*
*Pfdhfr*	RES108B	108B	WT or MutA	MutB	*
*Pfdhfr*	RES164	164	WT	MUT	*
*Pfdhfr*	RES164B	164B	WT	MUT	*
*Pfdhps*	RES436	436	WT	Mut1	
*Pfdhps*	RES437	437	MUT	WT	
*Pfdhps*	RES540	540	WT	MUT	
*Pfdhps*	RES581	581	WT	MUT	
*Pfdhps*	RES613	613	MutA	WT or MutB	
*Pfdhps*	RES613B	613B	WT or MutA	MutB	
*Pfdhps*	RES640	640	MUT	WT	
*Pfdhps*	RES645	645	MUT	WT	
*Pfmdr1*	RES86	86	WT	MUT	*
*Pfmdr1*	RES184	184	WT	MUT	*
*Pfmdr1*	RES1034	1034	WT	MUT	*
*Pfmdr1*	RES1042	1042	MUT	WT	*
*Pfmdr1*	RES1246	1246	MUT	WT	*
*Pfcrt*	RES72	72	MUT	WT	*
*Pfcrt*	RES74	74	MUT	WT	*
*Pfcrt*	RES75	75B1	WT	MUT	*
*Pfcrt*	RES76	76	WT	MUT	*
*Pfcrt*	RES97	97	WT	MUT	*
*Pfcrt*	RES152	152	WT	MUT	
*Pfcrt*	RES163	163	WT	MUT	
*Pfcrt*	RES220	220	MUT	WT	
*Pfcrt*	RES271	271	WT	MUT	
*Pfcrt*	RES326	326	WT	Mut1	
*Pfcrt*	RES326B	326B	Mut2	WT	
*Pfcrt*	RES356	356	WT	Mut1	
*Pfcrt*	RES356B	356B	WT	Mut2	
*Pfcrt*	RES371	371	MUT	WT	
*PfATPase6*	RES538	538	WT	MUT	*
*PfATPase6*	RES574	574	MUT	WT	*
*PfATPase6*	RES623	623	WT	MUT	*
*PfATPase6*	RES683	683	WT	MUT	*
*PfATPase6*	RES769	769	MUT	WT	*
*PfATPase6*	RES769B	769B	WT	MUT	*

(Mutations with * were sequenced and used for our comparison with the result of the two arrays).

**Table 2:** Comparison between ResMalChip and FlexiChip results.

**Gene Name**	**Agreement**	**Kappa (95% CI)**	**N**
*PfATPase6*	93.80%	0.862 (0.808 - 0.915)	387

*Pfmdr1*	98.26%	0.965 (0.935 - 0.995)	287

*Pfcrt*	94.12%	0.866 (0.829 - 0.902)	816

*Pfdhfr*	94.51%	0.890 (0.843 - 0.937)	364

*Pfdhps*	81.23%	0.612 (0.528 - 0.697)	341

Agreement: percentage of identical results, Kappa (95% CI): Kappa value with a 95% confidence interval, N: number of SNPs tested

### Direct sequencing of PCR products

A set of samples was sequenced for *Pfdhfr*, *Pfcrt*, *Pfmdr1 *and *PfATPase6*.genes. PCR products were purified using a P-100 Gel Fine solution (Biorad) and Multiscreen MAVN45 kit system (Millipore). Sequencing reactions were performed on both strands using internal primers and ABI Prism BigDye Terminator chemistry. Sequencing reactions were run on ABI Prism 3100 Genetic Analyzer (Applied Biosystems) at the Plate-Forme Génomique of Institut Pasteur in Paris, and analysed with Seqscape software v.2.0. (Applied Biosystems).

### External quality control

Fifty *P. falciparum *isolates from Cambodia were tested blindly in Mahidol Oxford Research Unit according to their own protocols. Briefly five SNPs of *Pfmdr1 *(positions 86, 184, 1034, 1042 and 1246) and one SNP of *Pfcrt *genes (position 76) were screened with restriction length polymorphism methods [6,7]. The *Pfserca/Pfatpase6 *gene was sequenced (4068 bp). Results were compared to the four SNPs tested with FlexiChip.

## Results

### Comparison between ResMalChip, FlexiChip and sequence results

#### ResMalChip

Twenty five gpr files generated by the Axon GenePix^® ^Pro software were analysed. They included data from 10520 SNPs corresponding to 263 samples tested for 40 positions on five genes. The best compromise between the number of ambiguity rejections and the number of misclassifications was obtained with an optimal rejection threshold of C_r _= 0.2. On the 10520 SNPs data handled by this algorithm, 1396 (13.3%) were classified as "weak signal" and 905 (8.6%) were rejected for ambiguity (they belong to ω_0_, the intermediate class between the "red" and "green" classes). Among the 1642 SNPs data which could be compared to the sequence, 218 (13.3%) were classified as "weak signal" and 109 (6.6%) "indeterminate" (inconsistency between replicates or ambiguity). Compared to sequencing, considered the "gold standard", a good agreement was found with 96.63% and 95.74% for sensitivity and specificity respectively.

#### FlexiChip

Six gpr files were analysed corresponding to 5000 SNPs data from 125 samples (part of the previous 263 samples analysed within ResMalChip) tested for 40 positions on five genes. The optimal rejection threshold value C_r _was also 0.2. On 5000 SNPs analysed by the algorithm, 332 (6.6%) were classified as "weak signal" and 222 (4.4%) "indeterminate", *i.e*. two to three times less than with the ResMalChip array. Among the 1215 SNPs data for which the sequences were available, 28 (2.3%) and 38 (3.1%) were respectively considered as "weak signal" and "indeterminate". Sensitivity and specificity were 95.88% and 97.68% respectively.

#### ResMalChip versus FlexiChip

A total of 3,078 SNP data corresponding to 81 samples and 38 positions on five genes were available in both ResMalChip and FlexiChip datasets. Among them, 2195 SNPs data were interpretable ("red" or "green" signal) with both techniques. An identical diagnosis was found for 2,034 (92.7%) of the SNP data (kappa coefficient 0.8491 with a standard error of 0.0213). When the results on a gene-by-gene basis were considered (Table 2), a very good agreement was found for *dhfr*, *crt*, *atpas*e and *mdr1 *gene. The main discrepancies were observed for the *dhps *gene.

### Comparison between FlexiChip and Mahidol-Oxford Research Unit (MORU) results

Fifty isolates were tested for eight SNPs in parallel in MORU with standard methods and with the FlexiChip. Among the 400 SNP data, 34 (8.5%) were classified as "weak signal" or "indeterminate". Among the 366 remaining SNP data, results were identical with both techniques in 365 cases (99.7% specificity, 91.5% sensibility, kappa coefficient 0.9923 with a standard error of 0.0523).

## Discussion

Molecular tools are essential for monitoring emergence and spread of anti-malarial drug resistance and are part of strategies described by the World Wide Anti-malarial Resistance Network (WWARN) consortium [22,23]. Correlation of molecular markers with in vivo and in vitro drug resistance has been clearly established for *dhfr/dhps *(sulphadoxine-pyrimethamine) and *pfcrt *(chloroquine) mutations, *mdr1 *(chloroquine, mefloquine) and *cytochrome b *(atovaquone). The microarray method described in this paper enables to implement molecular monitoring on a large scale because of the possibility to automatically analyse and interpret the results.

The aim of this project was to evaluate the flexible microarray under practical conditions using field isolates, in which multiple infections are frequently observed. Without any dye bias on the array, spots associated with mixed alleles should exhibit a "yellow" signal corresponding to a mix between red and green signals. In the framework of the proposed mathematical model, these spots should then fall in the intermediate class ω_0_. Thus, this class would be used to detect mixed infections instead of indeterminate ones. However, this mathematical property of the model could not be fully validated for several reasons. First, in the current study some SNPs showed no polymorphism in the processed samples. Indeed, field samples were sequenced for 20 SNPs out of 40 that were genotyped on the array. Among these 20 sequenced SNPs, only five showed polymorphism, with only one having both alleles in (almost) equal amount. Therefore, any dye bias on the signals measured on FlexiChip cannot be excluded, it would prevent mixed signals to behave as expected by the mathematical model. Second, the gold standard used to compare FlexiChip results with is sequencing. This method may not be the best one in the case of mixed alleles because chromatograms may be difficult to interpret, leading to erroneous sequences. The parameters of the mathematical model are derived on an array-by-array basis in order to adapt to possible technical variabilities between arrays. So they depend also on the proportion of single and mixed infections that are hybridized on the array. As most of the field samples analysed in this study were not polymorphic, the model behaviour in the case of a majority of mixed alleles cannot be predicted. But it is doubtless that it will have to be adapted to match the data distribution in that particular case. Finally, the actual design of FlexiChip makes it non exhaustive, as the use of two colours only for most of the monitored SNPs makes it unable to detect all the mutations The use of two mix combinations for the SNP located at position 108 on the *pfdhfr *gene led to a good classification rate of 100%. It is clear that extending the concept to the whole set of SNPs would increase the reliability of the base calling process, even in the case of mixed infections. Nevertheless, ResMalChip microarray has already been used in an environment of complex malaria infections like [24].

Combined with the FlexiChip microarray, the software provided a sensitivity and specificity of 95.88% and 97.68% respectively when compared to sequencing as the reference method. Moreover, the method performed well when compared to results obtained in a reference laboratory, with 99.7% concordance (kappa coefficient 0.9923 with a standard error of 0.0523).

The proposed package can be useful for epidemiological surveys and can give information on the dynamics of emergence and spread of genetic markers in time and/or in space. However, the method cannot be used as an immediate diagnostic tool for individual samples, because the format requires a high number of samples tested at one time to be cost effective.

In contrast to previous methods, FlexiChip is no longer dedicated to a single set of genes and/or organisms. Thanks to its flexibility, integration of new SNPs linked to anti-malarial drug resistance is made simpler and adjunction of species identification is now possible. It is easy to adapt to other loci and in particular for SNP detection of other organisms like HIV or Multi Drug Resistant Tuberculosis strains. Moreover, FlexiChip package is ready for use and adaptable to large scale studies to validate new molecular marker candidates.

## Concluding remarks

One of the major obstacles for implementation of molecular monitoring of resistance lies in the absence of practical tools for high throughput analysis. Universal microarrays such as FlexiChip could help to change this, as they are adapted to processing of numerous samples and easily adaptable to new markers. Furthermore, they are well suited for molecular biology laboratories from endemic countries, which need a robust and simple tool that could be easily adapted to a specific epidemiological situation.

## Competing interests

The authors declare that they have no competing interests.

## Authors' contributions

NS, AC, JYC, OMP, HPB and FA were involved in the conception and design of the microarray, MAD developed the software, NS, NK, OS, SC, PL, CB, MI, AMD, DS managed experimental procedure and performed field and laboratory work, NS, MAD, CR and FA participated in the statistical analysis, NS, MAD, CR, JYC and FA drafted and critically revised the manuscript. All authors read and approved the manuscript.

## Supplementary Material

Additional file 1 **List of cZip Codes spotted on FlexiChip**. *Table S1*: List of cZip Codes spotted on FlexiChip. *: discarded from the analysis (number 49 and 61) Click here for file

Additional file 2 **Scheme of the spotted oligonucleotides and expected hybridization**. *Table S2*: Scheme of the spotted oligonucleotides and expected hybridization. A) Spotting pattern of cZip on FlexiChip (Neg = Spot of water; Cy3 and Cy5 = anchor oligonucleotides prelabeled with Cy3 or Cy5). B) Example of a FlexiChip within SNPs associated with parasite resistance to anti-malarial drugs used (blue and white areas correspond to the two samples that can be diagnosed at the same time; zip41 to 48 and 89 to 96 are not used on this microarray) Click here for file

## References

[B1] Ariey F, Fandeur T, Durand R, Randrianarivelojosia M, Jambou R, Legrand E, Ekala MT, Bouchier C, Cojean S, Duchemin JB, Robert V, Le Bras J, Mercereau-Puijalon O (2006). Invasion of Africa by a single *pfcrt *allele of South East Asian type. Malar J.

[B2] WHO (2007). Methods and techniques for clinical trials on antimalarial drug efficacy: genotyping to identify parasite populations. Informal consultation organized by the Medicines for Malaria Venture and cosponsored by the World Health Organization.

[B3] Djimde A, Doumbo OK, Steketee RW, Plowe CV (2001). Application of a molecular marker for surveillance of chloroquine-resistant falciparum malaria. Lancet.

[B4] Wang X, Mu J, Li G, Chen P, Guo X, Fu L, Chen L, Su X, Wellems TE (2005). Decreased prevalence of the *Plasmodium falciparum *chloroquine resistance transporter 76T marker associated with cessation of chloroquine use against *P. falciparum *malaria in Hainan, People's Republic of China. Am J Trop Med Hyg.

[B5] Felger I, Beck HP (2002). Genotyping of *Plasmodium falciparum*. PCR-RFLP analysis. Methods Mol Med.

[B6] Duraisingh MT, Jones P, Sambou I, von Seidlein L, Pinder M, Warhurst DC (2000). The tyrosine-86 allele of the *pfmdr1 *gene of *Plasmodium falciparum *is associated with increased sensitivity to the anti-malarials mefloquine and artemisinin. Mol Biochem Parasitol.

[B7] Djimde A, Doumbo OK, Cortese JF, Kayentao K, Doumbo S, Diourte Y, Dicko A, Su XZ, Nomura T, Fidock DA, Wellems TE, Plowe CV, Coulibaly D (2001). A molecular marker for chloroquine-resistant falciparum malaria. N Engl J Med.

[B8] Rason MA, Andrianantenaina HB, Ariey F, Raveloson A, Domarle O, Randrianarivelojosia M (2007). Prevalent *pfmdr1 *N86Y mutant *Plasmodium falciparum *in madagascar despite absence of *pfcrt *mutant strains. Am J Trop Med Hyg.

[B9] Price RN, Uhlemann AC, Brockman A, McGready R, Ashley E, Phaipun L, Patel R, Laing K, Looareesuwan S, White NJ, Nosten F, Krishna S (2004). Mefloquine resistance in *Plasmodium falciparum *and increased *pfmdr1 *gene copy number. Lancet.

[B10] Lim P, Chy S, Ariey F, Incardona S, Chim P, Sem R, Denis MB, Hewitt S, Hoyer S, Socheat D, Merecreau-Puijalon O, Fandeur T (2003). *pfcrt *polymorphism and chloroquine resistance in *Plasmodium falciparum *strains isolated in Cambodia. Antimicrob Agents Chemother.

[B11] Juliano JJ, Trottman P, Mwapasa V, Meshnick SR (2008). Detection of the dihydrofolate reductase-164L mutation in *Plasmodium falciparum *infections from Malawi by heteroduplex tracking assay. Am J Trop Med Hyg.

[B12] Nair S, Brockman A, Paiphun L, Nosten F, Anderson TJ (2002). Rapid genotyping of loci involved in antifolate drug resistance in *Plasmodium falciparum *by primer extension. Int J Parasitol.

[B13] Rathod PK, Ganesan K, Hayward RE, Bozdech Z, DeRisi JL (2002). DNA microarrays for malaria. Trends Parasitol.

[B14] Gunderson KL, Steemers FJ, Lee G, Mendoza LG, Chee MS (2005). A genome-wide scalable SNP genotyping assay using microarray technology. Nat Genet.

[B15] Hirschhorn JN, Sklar P, Lindblad-Toh K, Lim YM, Ruiz-Gutierrez M, Bolk S, Langhorst B, Schaffner S, Winchester E, Lander ES (2000). SBE-TAGS: an array-based method for efficient single-nucleotide polymorphism genotyping. Proc Natl Acad Sci USA.

[B16] Crameri A, Marfurt J, Mugittu K, Maire N, Regos A, Coppee JY, Sismeiro O, Burki R, Huber E, Laubscher D, Puijalon O, Genton B, Felger I, Beck HP (2007). Rapid microarray-based method for monitoring of all currently known single-nucleotide polymorphisms associated with parasite resistance to antimalaria drugs. J Clin Microbiol.

[B17] Gerry NP, Witowski NE, Day J, Hammer RP, Barany G, Barany F (1999). Universal DNA microarray method for multiplex detection of low abundance point mutations. J Mol Biol.

[B18] Kalendar R, Lee D, Schulman AH (2009). FastPCR Software for PCR Primer and Probe Design and Repeat Search. Genes, Genomes and Genomics.

[B19] R Development Core Team (2007). R: A language and environment for statistical computing. R Foundation for Statistical Computing, Vienna, Austria.

[B20] Smyth GK, Speed TP (2003). Normalization of cDNA microarray data. Methods.

[B21] Fraley C, Raftery AE (2006). MCLUST Version 3 for R: Normal Mixture Modeling and Model-Based Clustering, Technical Report no. 504, Department of Statistics, University of Washington.

[B22] Plowe CV, Roper C, Barnwell JW, Happi CT, Joshi HH, Mbacham W, Meshnick SR, Mugittu K, Naidoo I, Price RN, Shafer RW, Sibley CH, Sutherland CJ, Zimmerman PA, Rosenthal PJ (2007). World Antimalarial Resistance Network (WARN) III: molecular markers for drug resistant malaria. Malar J.

[B23] Sibley CH, Barnes KI, Plowe CV (2007). The rationale and plan for creating a World Antimalarial Resistance Network (WARN). Malar J.

[B24] Ibrahim ML, Steenkeste N, Khim N, Adam HH, Konate L, Coppee JY, Ariey F, Duchemin JB (2009). Field-based evidence of fast and global increase of *Plasmodium falciparum *drug-resistance by DNA-microarrays and PCR/RFLP in Niger. Malar J.

