# Supplementary material for: FlexiChip package: an universal microarray with a dedicated analysis software for high-thoughput SNPs detection linked to anti-malarial drug resistance
Source: Malar J. 2009 Oct 15;8:229. doi: 10.1186/1475-2875-8-229 (PMC2770542; doi:10.1186/1475-2875-8-229)
Supplement: Additional file 1 — List of cZip Codes spotted on FlexiChip. Table S1: List of cZip Codes spotted on FlexiChip. *: discarded from the analysis (number 49 and 61) [file 1475-2875-8-229-S1.DOC]

**Additional figure 1:**

| ID | 5' cZip 3' cccccccNH2 | ID | 5' cZip 3’ cccccccNH2 | ID | 5' cZip 3' cccccccNH2 |
| --- | --- | --- | --- | --- | --- |
| 1 | GGGAGAGCGCAGCAGGCAACAGAG | 33 | ACGAGGCACACAGCCACACGCGAC | 65 | CGCGAGAAGGAGGGACGAGCCACA |
| 2 | GACCGCCACCAAGAACAGCACCGG | 34 | CAAGGCACCCCAGAAGAGACCGCC | 66 | CCACAGAGAGCACGGACCGACACC |
| 3 | GCGCCAACGCAGACCGGAAGACCA | 35 | AAGCCCAGCCCAGCCGAACGGAGA | 67 | CAGACCCACGCAGGGCGGGAGAAA |
| 4 | CGCGCACAGAAGGGCGAGAGACGA | 36 | CGGCCCCCGGAACAAGACAGACAC | 68 | AAAGCGCCAGGGCAGGACCCGAAG |
| 5 | GCAGGGAAGCGGGAGCGAAACAGC | 37 | AGCGGCAACGGACGCCAAGACACC | 69 | AGAGGCGCCCGCGCGAAGCAGAAA |
| 6 | AAAACGGGGCACAGCGCGGCGGAA | 38 | GGAAACCGACCCGCCCCGACACAA | 70 | GGACGGGCCCGAGGAAACACGCAA |
| 7 | CCCCGAGAACGCCCGAAGCACAAG | 39 | CGCCCGGAGAAGAAGCGCGAGCAA | 71 | CCGGCGAACGAAGAACCCGAACCG |
| 8 | GGGAACGACACAGACAAGCCGGGG | 40 | GGGGAGCCCCAACCCCAACAAAGC | 72 | GGCCACAACCCACAACGAGGACCG |
| 9 | GGCGGGAACCGAAGACAGGGGAAG | 41 | CGAAGGACCACAAGGGCCACGACG | 73 | CACGGCGGGGACAGAAAGGACCAC |
| 10 | GACAACGGGCAGCGACCGGACCAA | 42 | CACGAAAGCGAAGAGGACCGCGCC | 74 | AGAGAAGCCGACGAGGGCAGCACG |
| 11 | CCCAAAGCCCGCAACCCGACCAAC | 43 | CCACAAACACGGGGGCGACGACGA | 75 | CGAAGCCAAACCGGGCAACACCGG |
| 12 | CAGGAACCAAGCCAGCCAGAGGCC | 44 | CCGCGCAGGCGAAAGGAAGGCAGA | 76 | AAACCCCGGCCAACAGCCCCGCAA |
| 13 | ACACCACAGGACACACGCCCCAGG | 45 | AGCGCGCCCACACCAGCGAAAAGC | 77 | CGAGCGAGACACGGAACGGACACG |
| 14 | AGGCGCACCGCAAGACGAAACGGG | 46 | CGACCGAACCCCCACCGACAGGAA | 78 | ACAACAACGCGCCGCGGGAAAGGC |
| 15 | CACGCGCAACACGCAACACCCAGC | 47 | GGACCGGGAAAGAGCAGGAGACCG | 79 | ATGCCTCGCGCGGCGACAAGGATC |
| 16 | CACGAGGACGGCGACGAACCAGGA | 48 | AGAAACGCGGGCGGACCCAGGAAG | 80 | GGAAAGTTGTCGTCGTCCGCGCCC |
| 17 | AACGGCCGACCGCGGACGAACAGA | 49* | ACCGCCGCAAAACACCGGACGGCA | 81 | CACGCGCTGTAACGAGGGCGCAGA |
| 18 | CGGAGGGCGCAAACGACGACAGAC | 50 | GCGGCAGGGGGACCAGCAAACCAA | 82 | CCCGTGTGATGCGGAACTGTGCGC |
| 19 | GACGACGGAAGGGGACGCACAGCA | 51 | GAAGCCCCCAAGCGAGCCAGGAGA | 83 | ACGGGGTGACATCCGGCAAACCCG |
| 20 | CAACCACCAGACAGCGGACACGCG | 52 | AAACGGCGCGGAGACAACCGGAGC | 84 | TGAGGGTCCACATGAGTCGGGCCG |
| 21 | CCCAAGGGAGGAAGGCCGGGGAAA | 53 | ACGGCCCAAAACGCCACGGCAAGC | 85 | GCGACCCCCCATAGCGTGACCCAT |
| 22 | GAGGCCAAAGAGAACCGCGACGGG | 54 | GGCCGAGAGCCAAGGCCAGAGACA | 86 | TGCCGCTCGGCCATTCGCAGATCG |
| 23 | GACAACAGGCCGCCGAAGGGCAAG | 55 | AGACAAAACCGCACGCGGCCGGAG | 87 | CACGATCCCTCCGCTATCGGCCCT |
| 24 | CGGCACGACCCCACGAGAACAGAG | 56 | GCGGGGGGCAAAAACCAGACCACG | 88 | AGCAGCCTTGTCCCCCTGCCTGAC |
| 25 | GCCCCCCGAAAGAACGAGACCCCA | 57 | CAAGCAACGACCAGAGCCCAGGCC | 89 | CAAGAGGCGATACAGGCGCAGGGG |
| 26 | ACAAGCGGAACCCAGACCACGCGC | 58 | ACGCACCCGCAGAGGGGAGACGAA | 90 | CGAAGTCCTGGCCCCTTGCCTCAC |
| 27 | ACCAGGGAGACGGGACAAAGCGGC | 59 | AAAAGACGCCGAGGCGGGCGGCAA | 91 | GGGAACGGGCAGTCAGAACGTGGG |
| 28 | AGCAGCCAACCGGCAAGGGACCGA | 60 | GACAGCAAGGCGACACGACCGAGG | 92 | TTGGTGTTGAGCGCCAGCCCGACC |
| 29 | AACCCGGCGGCGAGGGAAACGACA | 61* | AGACGGACAGCCGCCAGCACGAAG | 93 | GCAGTGGCGCCTAGTGGATGCCCA |
| 30 | GCACGCCGCCCAAGAGCGAAAACC | 62 | AACAGACGCGAGGAGGAGGCACGC | 94 | GCCCCAACCGTGCAGCACTCCTCA |
| 31 | GGAGCCGCACCACGGCAGCAACAA | 63 | AGCCGGCACAAAAGGCCCGCAAGG | 95 | GCGGGTTACCGATCTGCTACGCCG |
| 32 | GGGGCAGAACACAGGCGGAACCCA | 64 | GCACCGAGCACACGGCCACCAAAG | 96 | GTGACGGCTGGAGTGCCGACGGAA |
