# Supplementary material for: FlexiChip package: an universal microarray with a dedicated analysis software for high-thoughput SNPs detection linked to anti-malarial drug resistance
Source: Malar J. 2009 Oct 15;8:229. doi: 10.1186/1475-2875-8-229 (PMC2770542; doi:10.1186/1475-2875-8-229)
Supplement: Additional file 2 — Scheme of the spotted oligonucleotides and expected hybridization. Table S2: Scheme of the spotted oligonucleotides and expected hybridization. A) Spotting pattern of cZip on FlexiChip (Neg = Spot of water; Cy3 and Cy5 = anchor oligonucleotides prelabeled with Cy3 or Cy5). B) Example of a FlexiChip within SNPs associated with parasite resistance to anti-malarial drugs used (blue and white areas correspond to the two samples that can be diagnosed at the same time; zip41 to 48 and 89 to 96 are not used on this microarray) [file 1475-2875-8-229-S2.DOC]

**Additional figure 2:**

Additional figure 2A

| Cy3 | Neg | Neg | Neg | CZip 1 | CZip 2 | CZip 3 | CZip 4 | CZip 5 | CZip 6 | CZip 7 | CZip 8 | CZip 9 |
| --- | --- | --- | --- | --- | --- | --- | --- | --- | --- | --- | --- | --- |
| cZip 10 | cZip 11 | cZip 12 | cZip 13 | cZip 14 | cZip 15 | cZip 16 | cZip 17 | cZip 18 | cZip 19 | cZip 20 | cZip 21 | cZip 22 |
| cZip 23 | cZip 24 | cZip 25 | cZip 26 | cZip 27 | cZip 28 | cZip 29 | cZip 30 | cZip 31 | cZip 32 | cZip 33 | cZip 34 | cZip 35 |
| cZip 36 | cZip 37 | cZip 38 | cZip 39 | cZip 40 | cZip 41 | cZip 42 | cZip 43 | cZip 44 | cZip 45 | cZip 46 | cZip 47 | cZip 48 |
| cZip 49 | cZip 50 | cZip 51 | cZip 52 | cZip 53 | cZip 54 | cZip 55 | cZip 56 | cZip 57 | cZip 58 | cZip 59 | cZip 60 | cZip 61 |
| cZip 62 | cZip 63 | cZip 64 | cZip 65 | cZip 66 | cZip 67 | cZip 68 | cZip 69 | cZip 70 | cZip 71 | cZip 72 | cZip 73 | cZip 74 |
| cZip 75 | cZip 76 | cZip 77 | cZip 78 | cZip 79 | cZip 80 | cZip 81 | cZip 82 | cZip 83 | cZip 84 | cZip 85 | cZip 86 | cZip 87 |
| cZip 88 | cZip 89 | cZip 90 | cZip 91 | cZip 92 | cZip 93 | cZip 94 | cZip 95 | cZip 96 | Neg | Neg | Neg | Cy5 |

Additional figure 2B

| Cy3 | Neg | Neg | Neg | Pfdhfr-16 | Pfdhfr-51 | Pfdhfr-59 | Pfdhfr-108 | Pfdhfr-108B | Pfdhfr-164 | Pfdhfr-164B | Pfdhps-436 | Pfdhps-437 |
| --- | --- | --- | --- | --- | --- | --- | --- | --- | --- | --- | --- | --- |
| Pfdhps-540 | Pfdhps-581 | Pfdhps-613 | Pfdhps-613B | Pfdhps-640 | Pfdhps-645 | Pfmdr1-86 | Pfmdr1-184 | Pfmdr1-1034 | Pfmdr1-1042 | Pfmdr1-1246 | Pfcrt-72 | Pfcrt-75B |
| Pfcrt-74 | Pfcrt-76 | Pfcrt-97 | Pfcrt-152 | Pfcrt-163 | Pfcrt-220 | Pfcrt-271 | Pfcrt-326 | Pfcrt-326B | Pfcrt-356 | Pfcrt-356B | Pfcrt-371 | PfATPase-538 |
| PfATPase-574 | PfATPase-623 | PfATPase-683 | PfATPase-769 | PfATPase-769B | Zip 41 | Zip 42 | Zip 43 | Zip 44 | Zip 45 | Zip 46 | Zip 47 | Zip 48 |
| Pfdhfr-16 | Pfdhfr-51 | Pfdhfr-59 | Pfdhfr-108 | Pfdhfr-108B | Pfdhfr-164 | Pfdhfr-164B | Pfdhps-436 | Pfdhps-437 | Pfdhps-540 | Pfdhps-581 | Pfdhps-613 | Pfdhps-613B |
| Pfdhps-640 | Pfdhps-645 | Pfmdr1-86 | Pfmdr1-184 | Pfmdr1-1034 | Pfmdr1-1042 | Pfmdr1-1246 | Pfcrt-72 | Pfcrt-75B | Pfcrt-74 | Pfcrt-76 | Pfcrt-97 | Pfcrt-152 |
| Pfcrt-163 | Pfcrt-220 | Pfcrt-271 | Pfcrt-326 | Pfcrt-326B | Pfcrt-356 | Pfcrt-356B | Pfcrt-371 | PfATPase-538 | PfATPase-574 | PfATPase-623 | PfATPase-683 | PfATPase-769 |
| PfATPase-769B | Zip 89 | Zip 90 | Zip 91 | Zip 92 | Zip 93 | Zip 94 | Zip 95 | Zip 96 | Neg | Neg | Neg | Cy5 |
